# Copper microsphere hybrid mesoporous carbon as matrix for preparation of shape-stabilized phase change materials with improved thermal properties

**DOI:** 10.1038/s41598-020-73114-z

**Published:** 2020-09-29

**Authors:** Yi Liu, Yan Chen, Junwei Zhang, Junkai Gao, Zhi Han

**Affiliations:** School of Naval Architecture and Maritime, Zhejiang Ocean University, Zhoushan, 316022 China

**Keywords:** Solar thermal energy, Energy storage

## Abstract

Copper microsphere hybrid mesoporous carbon (MPC-Cu) was synthesized by the pyrolysis of polydopamine microspheres doped with copper ions that were prepared using a novel, facile and simple one-step method of dopamine biomimetic polymerization and copper ion adsorption. The resulting MPC-Cu was then used as a supporter for polyethylene glycol (PEG) to synthesize shape-stabilized phase change materials (PEG/MPC-Cu) with enhanced thermal properties. PEG/MPC-Cu was studied by scanning electron microscopy, X-ray diffraction, Fourier transform infrared spectroscopy, X-ray photoelectron spectroscopy, thermogravimetric analysis, differential scanning calorimetry and thermal constant analysis. The results demonstrated that the thermal conductivity of PEG/MPC-Cu was 0.502 W/(m K), which increased by 100% compared to pure PEG [0.251 W/(m K)]. The melting enthalpy of PEG/MPC-Cu was 95.98 J/g, indicating that PEG/MPC-Cu is a promising candidate for future thermal energy storage applications. In addition, the characterization results suggested that PEG-MPC-Cu possessed high thermal stability. Therefore, the method developed in this paper for preparing shape-stabilized phase change materials with improved thermal properties has substantial engineering application prospects.

## Introduction

With the rapid growth of the economy, the imbalance between energy demand and production is worsening. The impending energy consumption crisis has encouraged further research into sustainable and renewable energy sources^1^. Renewable energy has gained attention due to its potential to address rising levels of greenhouse gas emissions and shortages of fossil fuels. Energy storage technologies are regarded as valid solutions to address issues with energy supply and demand and to improve energy utilization efficiency; in addition, these technologies are also effective strategies for environmental protection. Latent heat energy storage is an effective thermal energy storage method^2–4^ with high energy storage capacity^5^ and almost constant temperature during the phase transition process^6^.

Phase change materials (PCMs) are the media for latent heat energy storage and can absorb or release considerable heat during the phase transition^7^. PCMs have been widely investigated due to their high heat storage density, narrow working temperature and ability to store latent enthalpy at a given temperature, which is important in applications where a constant operating temperature is required^8,9^. In recent years, PCMs have been applied to many areas, such as energy-conserving buildings, waste heat recovery, and solar heating systems^10–12^. However, the wide applications of traditional PCMs were hindered by some flaws, such as low thermal conductivity, ease of leakage and changing volume in the process of phase change and the need for special latent devices, which increase the cost of usage and thermal resistance^13^. Therefore, in recent years, shape-stabilized PCMs (ss-PCMs) have been widely studied and discussed.

On the basis of the chemical compositions, PCMs are usually divided into organic and inorganic PCMs. Owing to the merits of favorable chemical and thermal stability, high latent heat density, suitable phase change temperature, low cost, and small temperature fluctuation^14–18^, organic PCMs are considered the most common materials used in latent heat storage. Among all organic PCMs, polyethylene glycol (PEG) has the advantages of a suitable phase transition temperature, high potential heat storage capacity, high chemical inertness and stability, low vapor pressure, nontoxicity and low cost^19^; thus, PEG is regarded as a promising organic PCM in the field of heat storage. Nevertheless, the drawbacks of leakage and poor thermal conductivity of PEG hinder its wide application in heat storage systems. In this work, PEG was used as the organic phase change material to synthesize ss-PCM.

To overcome the leakage problem of organic PCMs, some porous materials, such as mesoporous silica, diatomite, and porous carbon, were applied to support the synthesis of ss-PCMs from organic PCMs^20–22^. Chen et al*.*^20^ prepared wrinkled mesoporous silica (WMSN) and embedded myristic acid (MA) into WMSN to synthesize ss-PCMs, named MA/WMSN, which prevented the leakage problem of MA in the phase change process. Wan et al*.*^22^ synthesized ss-PCM using pinecone biochar (PB) as the matrix and palmitic acid (PA) as the organic PCM, which not only solved the leakage problem of PA but also improved the thermal conductivity of the PCMs. Apart from the leakage problem, organic PCMs have the disadvantage of low thermal conductivity. To address this issue, highly thermally conductive nanoparticles, such as metal nanoparticles, expanded graphite, graphene oxide and carbon nanotubes, were used as additives in the synthesis of shape-stabilized PCMs to enhance the thermal conductivity of ss-PCMs^23–26^. Cheng et al*.* utilized expanded perlite (EP) as the supporter for fixing synthesized tetradecanol (TD) to fabricate composite phase change materials of TD/EP, and in order to enhance the thermal conductivity, carbon fiber (CF) and Cu powder (CuP) were added into ss-PCMs. The thermal conductivities of TD-CuP/EP and TD-CF/EP were enhanced approximately two times compared to that of TD/EP^23^. Lu et al. used polyethylene glycol (PEG)-based polyurethane (PU) as the organic PCM and wood powder (WP)/graphene oxide (GO) as the matrix to fabricate composite ss-PCMs of PEG-based PU/WP@GO. The GO nanosheet not only was a supporting material but also acted as a thermally conductive filler, and the thermal conductivity of PEG-based PU/WP@GO [1.87 W/(m K)] was much higher than that of PEG-based PU/WP without adding GO [0.3 W/(m K)]^26^. However, the preparation of the abovementioned nanoparticles with high thermal conductivity is a complex, expensive process with a potential to cause environmental pollution. Therefore, it is important to develop a preparation strategy for ss-PCMs with high thermal conductivity that is easy, inexpensive and environmentally friendly.

Inspired by the highly adhesive proteins secreted by mussels, Messersmith’s team developed a biomimetic functionalization strategy utilizing dopamine as the modifier^27,28^. Additionally, dopamine has been reported to turn into polydopamine microspheres by oxidization and to self-polymerized in alkaline solutions^29^. In this study, copper ions were introduced into an alkaline solution in which polydopamine microspheres (PDMS) were obtained by the simple method of biomimetic self-polymerization, and in the formation process of PDMS, the copper ions were absorbed by PDMS to form PDMS-Cu^2+^. Then, PDMS-Cu^2+^ was pyrolyzed, mesoporous carbon (MPC) with a high surface area and appropriate pore volume was obtained, and copper ions were reduced to copper powder. Finally, mesoporous carbon with Copper microspheres (MPC-Cu) was obtained.

In this paper, MPC-Cu was used as the supporter, and PEG was utilized as the organic phase change material to fabricate an efficient ss-PCM (PEG/MPC-Cu) by the vacuum impregnation method. The thermal energy storage properties of PEG/MPC-Cu and the interaction mechanism between PEG and MPC-Cu were studied. The results demonstrated that MPC-Cu can be used as an excellent organic phase change material matrix to prepare ssPCMs and that PEG/MPC-Cu with favorable phase change properties have great potential for latent heat storage applications.

## Experimental

### Materials and methods

PEG (average relative molecular mass: 4000) was obtained from Aladdin Industrial Corporation. Dopamine hydrochloride was purchased from Sigma-Aldrich. Cu (NO_3_)_2_∙3H_2_O was purchased from Sinpharm Chemical Reagent Co., Ltd.

### Preparation of MPC-Cu

PDMS with Cu^2+^ (PDMS-Cu^2+^) was synthesized. Briefly, 0.1 g Cu (NO_3_)_2_∙3H_2_O was dissolved in 200 ml phosphate buffer (pH = 7.5), and then 1 g dopamine hydrochloride was added into the above-treated phosphate buffer and stirred for 24 h in the dark. Next, the suspension was filtered and dried in an oven for 24 h at 45 ℃ to obtain PDMS-Cu^2+^. The pyrolysis of PDMS-Cu^2+^ was carried out in a tube furnace in a nitrogen atmosphere at a ramp rate of 3 ℃/min, and the highest temperature of 850 ℃ lasted for 2 h to obtain MPC-Cu.

### Preparation of PEG/MPC-Cu

**Figure 1:**
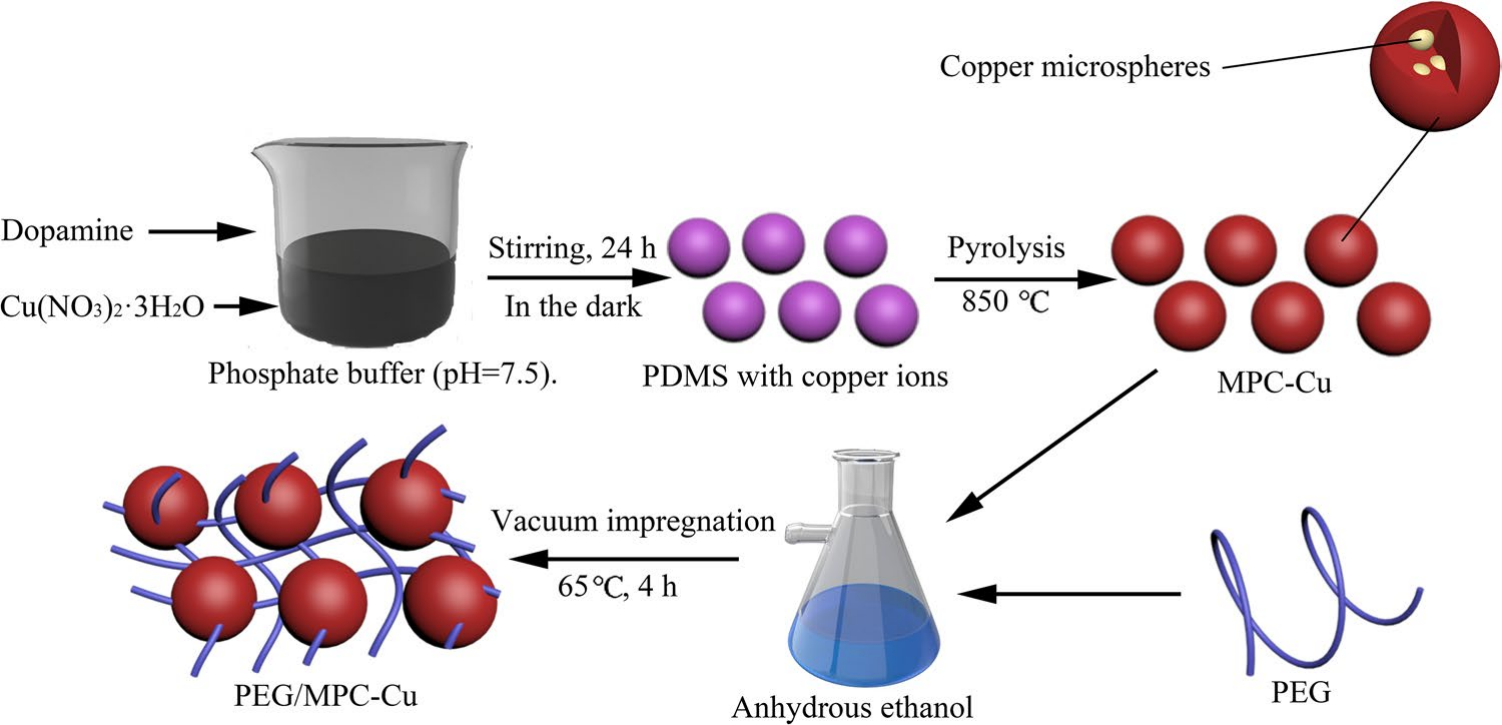
The preparation procedure for PEG/MPC-Cu.

The method applied in this study to prepare PEG/MPC-Cu was vacuum impregnation. Specifically, a certain mass of PEG was dissolved in 10 mL anhydrous ethanol, and then a certain mass of MPC-Cu was added to the as-prepared PEG solution. The mass ratio between PEG and MPC-Cu was 7:3. Then, the solutions were placed in a vacuum atmosphere for 1 h and stirred in a thermostat water bath at 65 ℃ for 4 h. Finally, the solutions were baked in a drying oven at 45 ℃. The obtained products were PEG/MPC-Cu composite PCMs. A sketch of the preparation procedure for PEG/MPC-Cu is displayed in Fig. 1.

### Characterization

Scanning electron microscopy (SEM, FEG-250, FEI, USA) was utilized to investigate the morphology of MPC-Cu and PEG/MPC-Cu. The pore properties were measured by using a Brunauer–Emmett–Teller analyzer (BET, Quanta, NOVA2000E, Boynton Beach, FL, USA) via N_2_ adsorption–desorption, which was carried out at − 196 ℃ under the relative vapor pressure range of 0.05–1, and the sample was degassed at 250 ℃ for 4 h. The chemical compatibilities of PEG, MPC-Cu and PEG/MPC-Cu were inspected by Fourier transform infrared spectroscopy (FT-IR, BrukerVECTOR22) at a wavenumber range of 400–4000 cm^−1^. The thermal stability of PEG, MPC-Cu and PEG/MPC-Cu was studied by a thermal gravimetric analyzer instrument (TGA, HCT-1, Beijing, China), and the operating temperature ranged from room temperature to 500 ℃ with a heating rate of 10 ℃/min. The crystalline structures of PEG, MPC-Cu and PEG/MPC-Cu were tested by X-ray diffraction (XRD, DX-2700, SHL-2, Thermal Scientific) at 40 kV and 30 mA. The scans were conducted in the 2θ range from 10° to 60°. X-ray photoelectron spectroscopy (XPS, ESCALAB 250Xi K-Alpha, Thermo Fisher) patterns for MPC-Cu and PEG/MPC-Cu were measured at 40 kV and 30 mA in the range from 10° to 60°. Differential scanning calorimetry (DSC) was utilized to observe the heat storage capacity of PEG, MPC-Cu and PEG/MPC-Cu by scanning differential calorimetry (DSC Q200) with a heating and cooling rate of 10 °C/min between 0 and 100 °C under a N_2_ atmosphere. A thermal constant analyzer (CTPS-2500) was applied to test the thermal conductivity of PEG and PEG/MPC-Cu.

## Results and discussion

### Characterization of MPC-Cu and PEG/MPC-Cu

**Figure 2:**
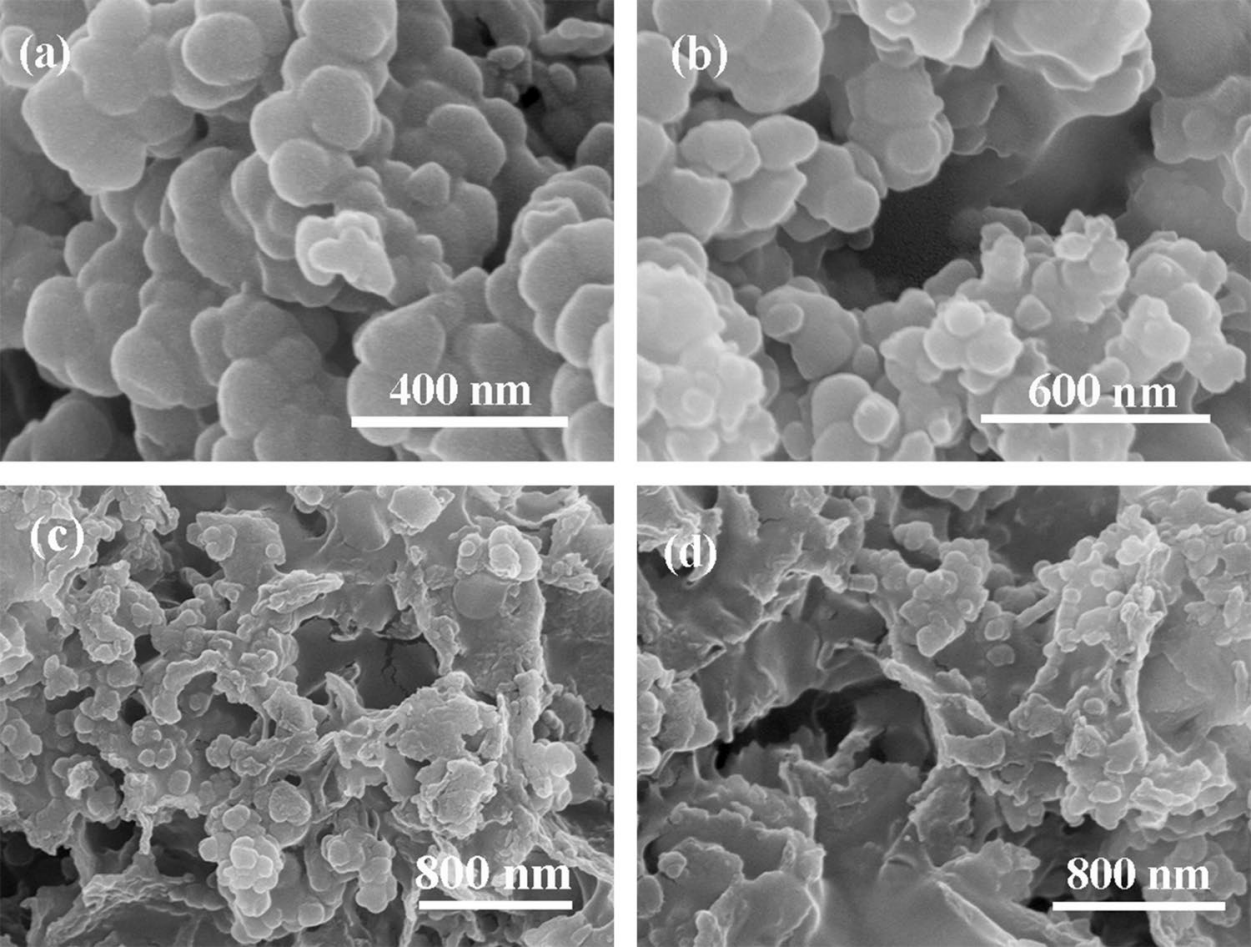
SEM images of MPC-Cu (**a**, **b**) and PEG/MPC-Cu (**c**, **d**).

The SEM images of MPC-Cu and PEG/MPC-Cu are shown in Fig. 2. As shown in Fig. 2a,b, MPC-Cu exhibited stacked spherical particles with rough surfaces, leading to the irregular edge of MPC-Cu. Figure 2c,d shows the SEM image of PEG/MPC-Cu. PEG/MPC-Cu exhibited a gel-like surface and particle structure, indicating that PEG was uniformly distributed on the surface of MPC-Cu.

### Pore properties of MPC-Cu

**Figure 3:**
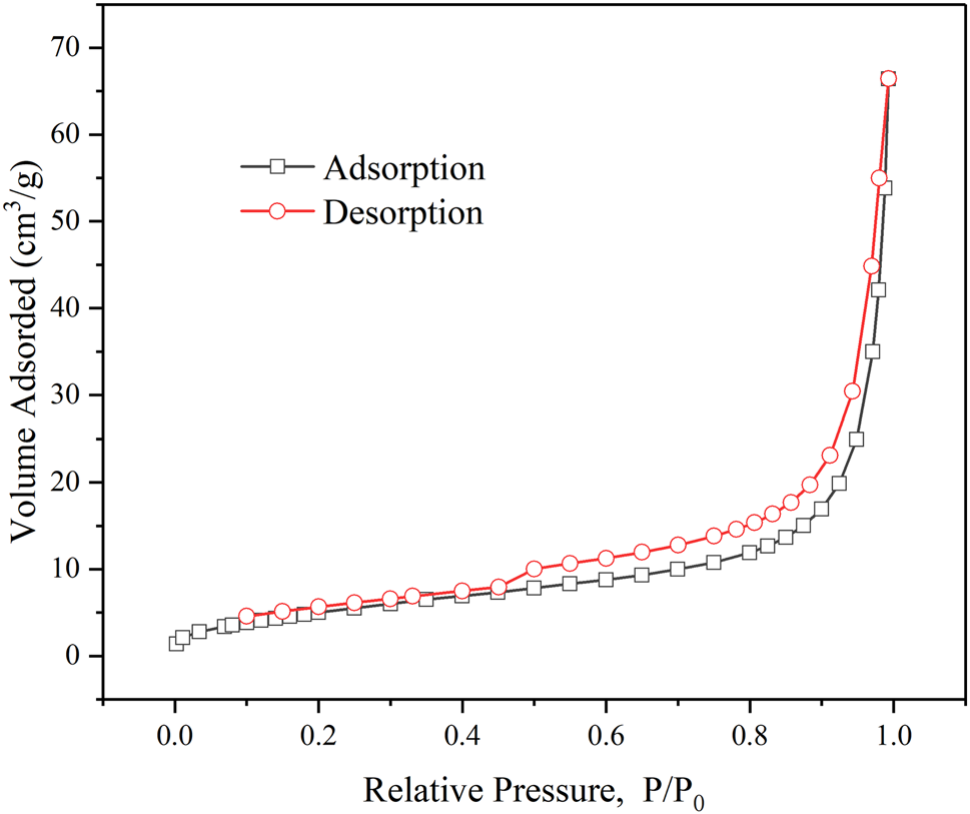
The nitrogen adsorption/desorption isotherms of MPC-Cu.

The nitrogen adsorption/desorption isotherms of MPC-Cu are displayed in Fig. 3, and the isotherms can be found classical type IV isotherms, corresponding to mesoporous material^30^. The BET surface area of MPC-Cu was 184.906 m^2^/g, and the BJH adsorption cumulative volume of MPC-Cu was 0.091 cm^3^/g. Furthermore, the calculated BJH pore diameter was 18.25 nm, which was beneficial to the adsorption of PEG and improved the stability of the as-prepared PCMs. In addition, the BET surface area of PEG/MPC-Cu was 8.428 m^2^/g, and its BJH adsorption cumulative volume and pore diameter were 0.00213 cm^3^/g and 2.5 nm, respectively, which were much smaller than that of MPC-Cu, indicating that PEG was successfully loaded into the aperture of MPC-Cu by vacuum impregnation.

**Figure 4:**
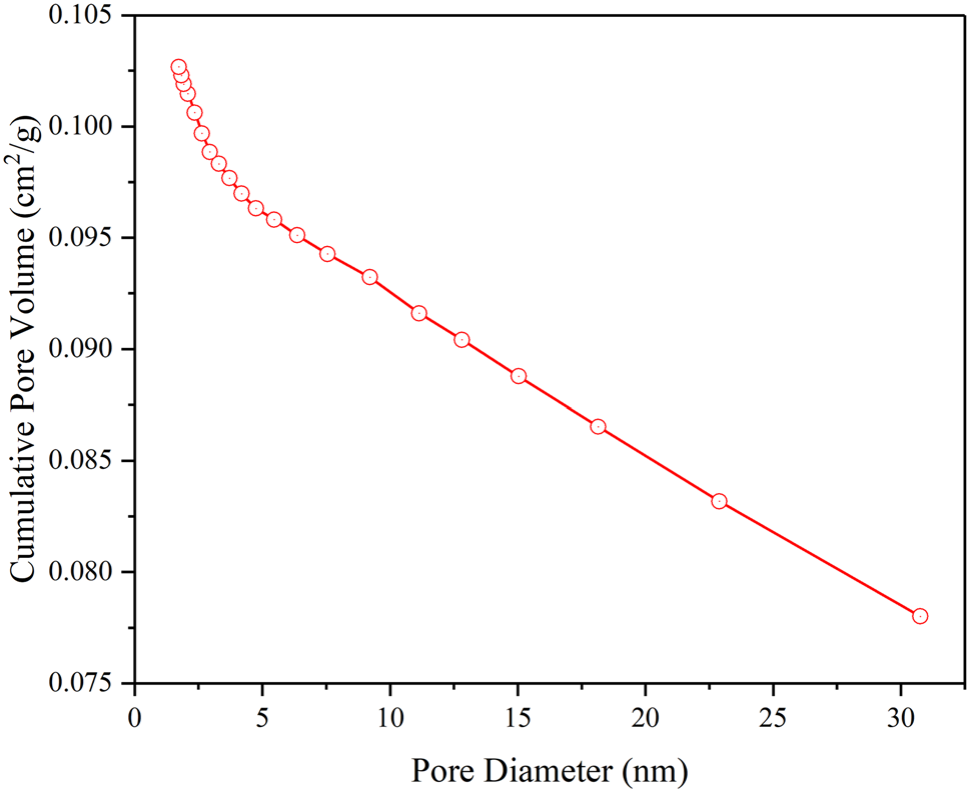
The pore diameter distribution of MPC-Cu.

Figure 4 displays the pore diameter distribution of MPC-Cu. The tested mean aperture was 18.25 nm, which again indicated that MPC-Cu was a kind of  mesoporous carbon material. These pore properties ensured that PEG could be embedded into the pores of MPC-Cu, and the stability of the as-prepared PEG/MPC-Cu could be enhanced.

### Chemical properties of PEG, MPC-Cu and PEG/MPC-Cu

**Figure 5:**
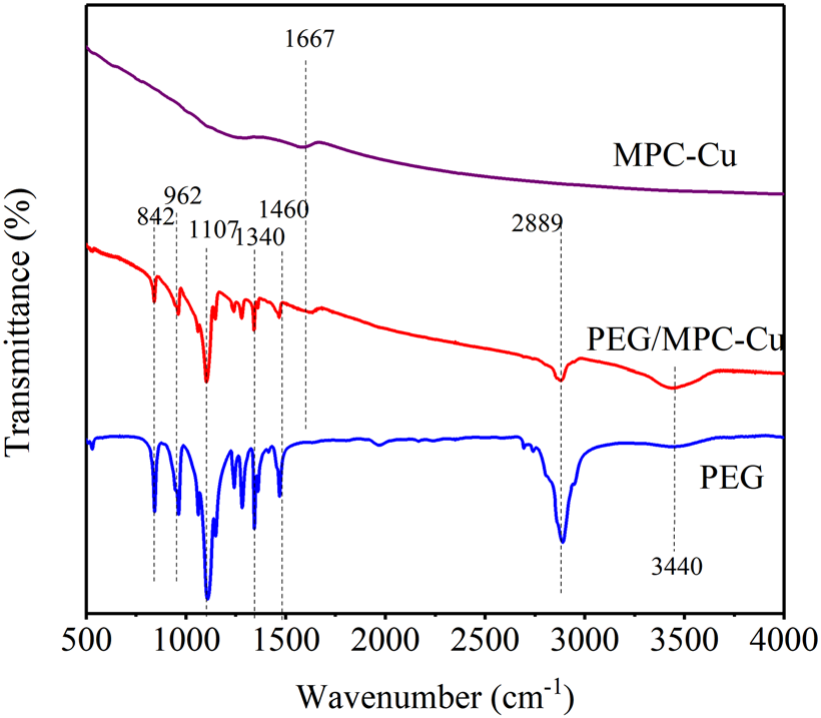
FI-IR spectra for PEG, MPC-Cu and PEG/MPC-Cu.

FI-IR was employed to investigate the chemical compatibility and chemical stability of MPC-Cu, PEG and PEG/MPC-Cu. Figure 5 shows the FT-IR spectra of MPC-Cu, PEG and MPC. Almost no significant peaks were observed in the curve for MPC-Cu, indicating that the pyrolysis did not add any new chemical functional groups to MPC-Cu. There was only a small broad absorption peak located at 1667 cm^−1^ because of the stretching vibrations of C=C^31^. For PEG, the strongest peak located at 1107 cm^−1^ was caused by the stretching vibration of C–O, and the asymmetric vibration of O–H resulted in the peak at 3440 cm^−1^^32^. The adsorption peaks at 842 cm^−1^, 962 cm^−1^, and 2889 cm^−1^ were attributed to asymmetrical and symmetrical stretching vibrations of –CH_2_^33^. The peaks at 1340 cm^–1^ and 1460 cm^−1^ resulted from the stretching vibration of C–H^34^. After fixing PEG on the carbon matrix, the curve for composite PCM (PEG/MPC-Cu) showed PEG absorption peaks, and no new peaks were observed. Therefore, there was no chemical interaction between PEG molecules and the matrix, indicating that MPC-Cu was a favorable carrier for PEG fixation, and the PCMs prepared with stable shapes had excellent chemical stability and compatibility^35,36^.

### Crystallization properties of PEG, MPC-Cu and PEG/MPC-Cu

**Figure 6:**
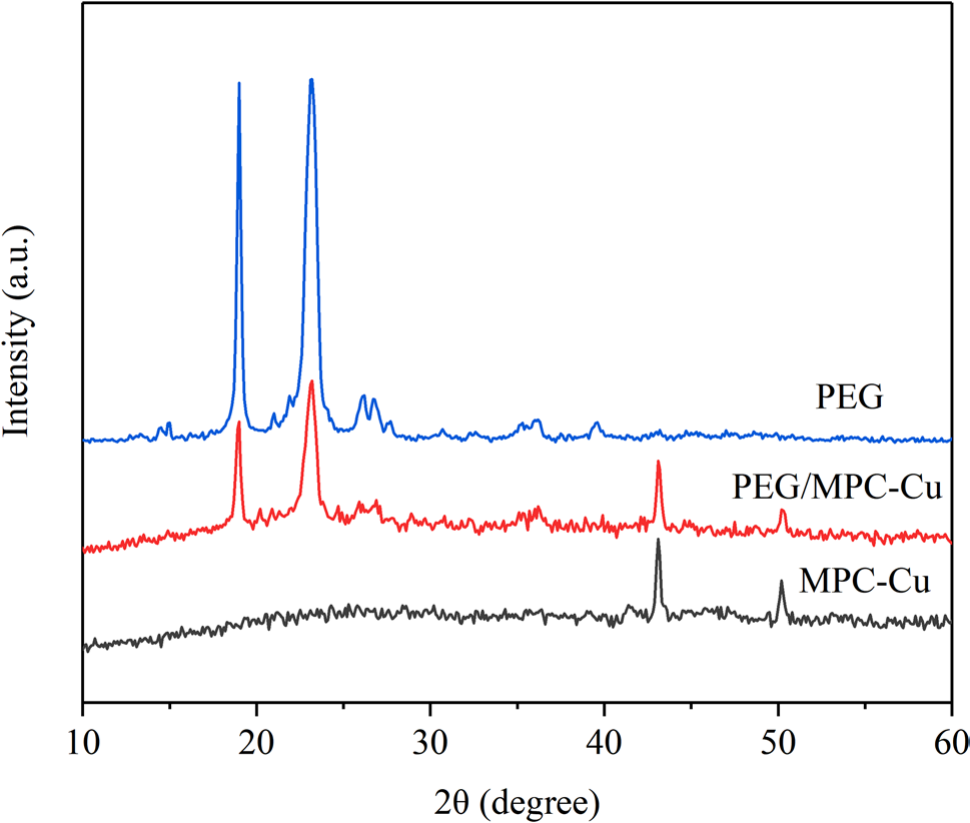
XRD patterns for PEG, PEG/MPC-Cu and MPC-Cu. a.u.: arbitrary unit.

XRD was conducted to determine the crystallization properties and chemical compatibilities of PEG, MPC-Cu and PEG/MPC-Cu, and the results can be seen in Fig. 6. In the pattern for PEG, there were two sharp diffraction peaks, which were located at approximately 19.0° and 23.2°, representing the {120} plane and {032} Miller plane, respectively^37^. Two diffraction peaks located at approximately 43.1° and 50.2° can be found in the pattern for MPC-Cu, which was attributed to the {111} and {200} crystal planes of the cubic copper monomer, respectively, demonstrating that the copper microspheres were successfully introduced into MPC^38^. Moreover, these diffraction peaks of PEG in PEG/MPC-Cu were slightly weaker than those of bare PEG because the capillary forces and van der Waals between PEG and MPC reduced the crystallinity of PEG in PEG/MPC-Cu^39,40^. In addition, the shapes of the patterns for PEG and PEG/MPC-Cu were similar, indicating that PEG was embedded in MPC-Cu without damaging its crystallization property and structure^41^. The similar shapes of the two patterns for PEG and PEG/MPC-Cu confirmed that the interaction between PEG and MPC-Cu was of physical nature, and therefore the chemical compatibility between the PEG molecules and MPC-Cu was favorable^42–44^.

### Surface element analysis of MPC-Cu and MPC

**Figure 7:**
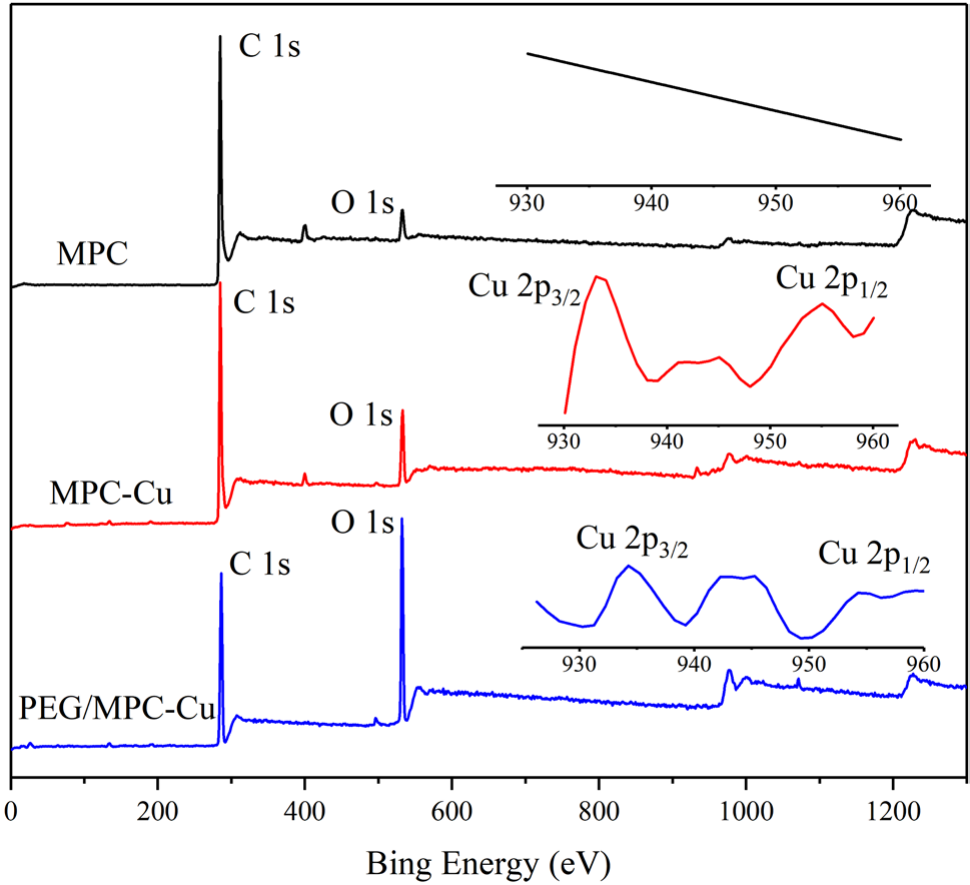
XPS survey spectra for MPC, MPC-Cu and PEG/MPC-Cu.

**Figure 8:**
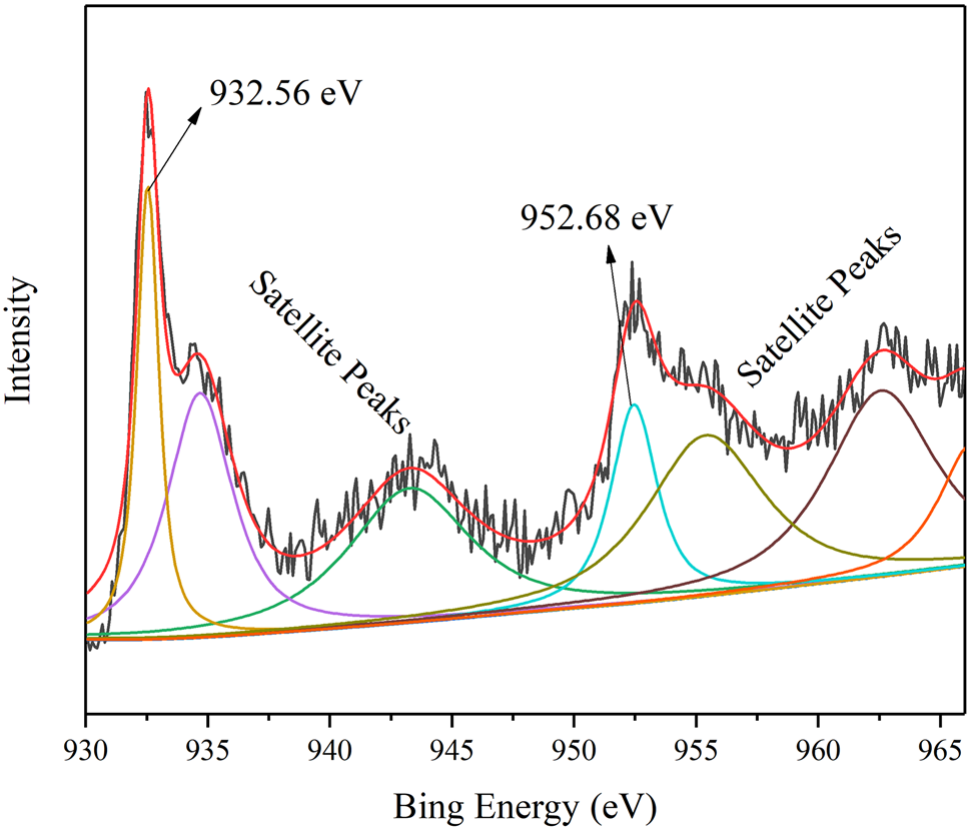
XPS pattern of MPC-Cu: Cu.

The elemental composition of MPC and MPC-Cu was determined by XPS, and the XPS curves are shown in Fig. 7. The locations of C1*s* in the spectra for MPC and MPC-Cu were 284.79 eV and 284.9 eV, respectively. The binding energies of O1*s* of MPC and MPC-Cu were 532.76 eV and 532.84 eV, respectively. In addition, two peaks at 932.56 eV and 952.68 eV appeared in the spectrum for MPC-Cu, which were attributed to Cu 2*p*_3/2_ and Cu 2*p*_1/2_, respectively^45–47^. Moreover, it was not smooth between the two peaks of Cu 2*p*_3/2_ and Cu 2*p*_1/2_, suggesting that some of the copper microspheres in the sample were oxidized^48^. Figure 8 shows the XPS analysis of copper element of MPC-Cu, and beside the two main peaks located at 932.56 eV and 952.68 eV (Cu 2*p*_3/2_ and Cu 2*p*_1/2_), there were also some satellite peaks in the XPS pattern of copper element of MPC-Cu. The satellite peaks at 934.68 eV and 943.38 eV were attributed to Cu 2*p*_3/2_ of CuO phase, and the satellite peaks at 955.48 eV was corresponding to Cu 2*p*_1/2_ of CuO phase^49^. According to the above study results, copper microspheres were successfully introduced into the carrier due to the appearance of the peaks of Cu 2*p*_3/2_ and Cu 2*p*_1/2_ in the MPC-Cu curves. Furthermore, it can be clearly seen that the O1*s* peak in the curve of PEG/MPC-Cu was much higher than that of MPC and MPC-Cu, which was because PEG contains O element, and the loading of PEG increased the oxygen content of PEG/MPC-Cu. Furthermore, the Cu 2*p*_3/2_ and Cu 2*p*_1/2_ peaks were obverted in the curve of PEG/MPC-Cu, and this result indicated that PEG/MPC-Cu contained copper microspheres, which could contribute to the improvement of the thermal conductivity of PEG/MPC-Cu.

### Thermal stability of PEG/MPC-Cu

**Figure 9:**
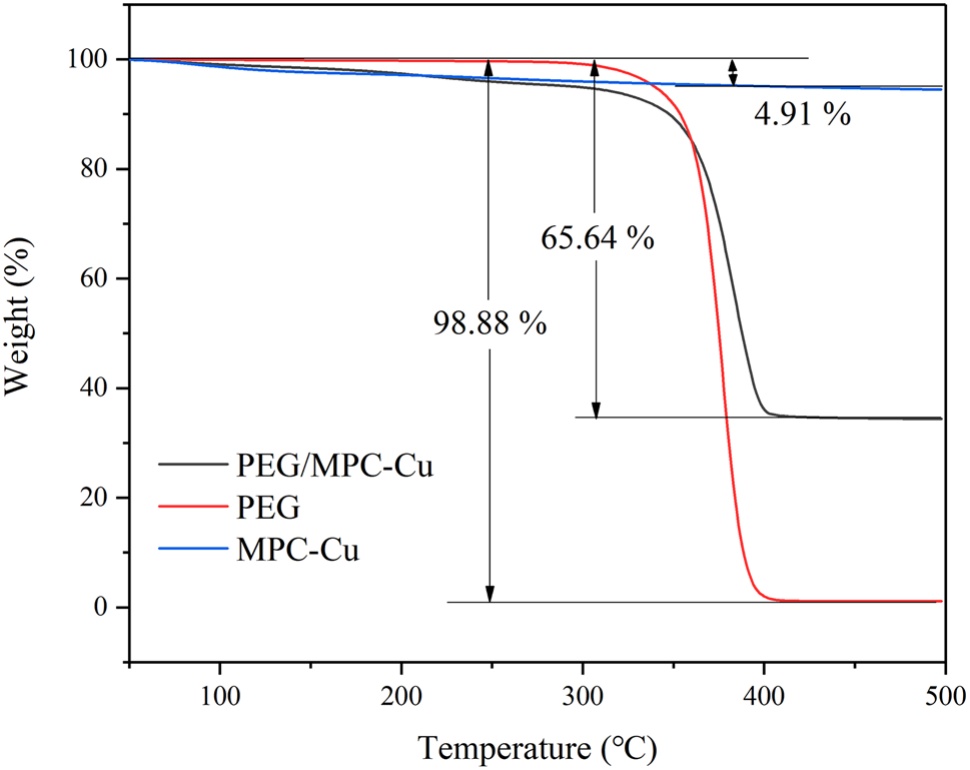
TGA curves for PEG, MPC-Cu and PEG/MPC-Cu.

Thermal stability is an important factor for evaluating the thermal regulation and thermal storage properties of composite PCMs. TGA was utilized to investigate the thermal stability of PEG, MPC-Cu and PEG/MPC-Cu. Figure 9 shows the TGA curves for PEG/MPC-Cu and pure PEG. As shown in the curves, there was no significant mass reduction for PEG when the temperature was below 300 ℃. However, the mass of PEG decreased sharply when the temperature was above 300 ℃, and at 400 ℃, the remaining mass was 1.12%, which was attributed to impurities in the PEG that was used in this study. For the TGA curve of MPC-Cu, the weight loss before 220 ℃ was 3.06%, which was attributed to the evaporation of water adsorbed on the surface or in the pores near the surface, and the weight loss from 220 to 400 ℃ was 1.85%, which was because of the volatilization of water adsorbed in the inner pores of MPC-Cu. Therefore, the total weight loss of MPC-Cu was 4.91%. Furthermore, approximately 10% weight reduction was observed in the curve for PEG/MPC-Cu when the temperature was below 350 ℃, and a 4% mass reduction occurred below 220 ℃. The mass reduction below 220 ℃ could be interpreted as evaporation of water inside or outside the pore^50^. There was a 6% mass reduction between 220 and 350 ℃ because with the increase in thermal conductivity, the thermal decomposition of PEG was accelerated, which reduced the decomposition temperature of PEG. The drastic weight reduction of PEG/MPC-Cu began at 350 ℃ and ended at 400 ℃, which was in correspondence with the thermal degradation of PEG in the supporter of MPC-Cu^51,52^. According to the study results, PEG/MPC-Cu exhibits high thermal stability and high temperature resistance.

### Thermal properties of pristine PEG and PEG/MPC-Cu composites

**Figure 10:**
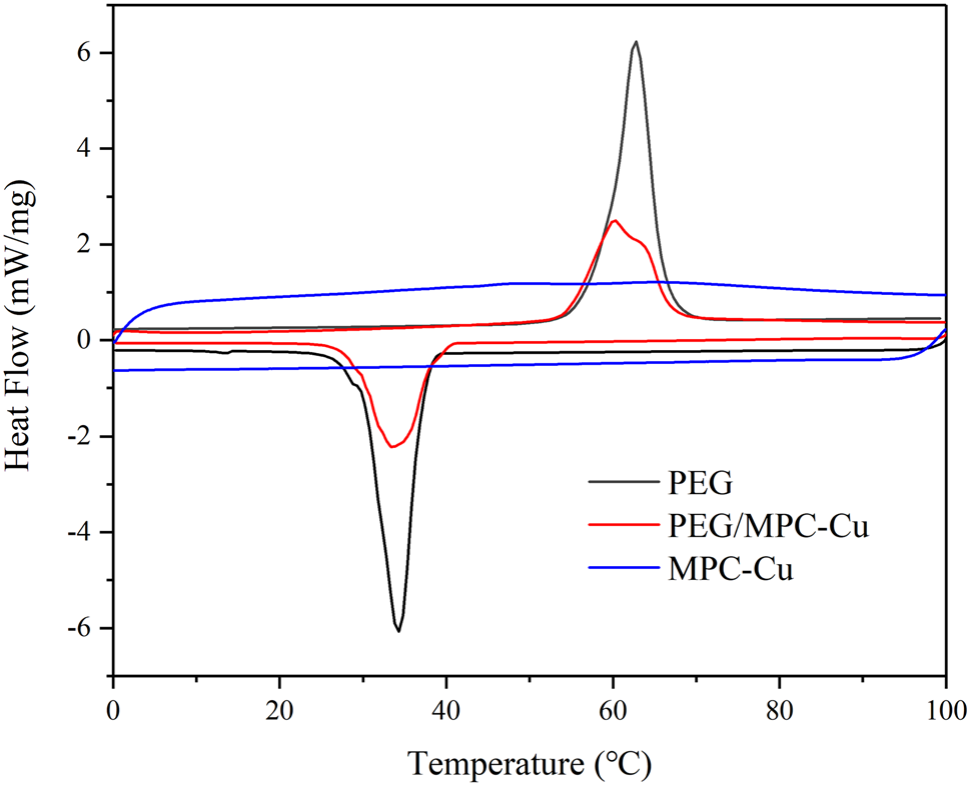
DSC curves for PEG, MPC-Cu and PEG/MPC-Cu.

The fusion and freezing enthalpies play a significant role in the practical application of ss-PCMs, and the higher the fusion and freezing enthalpies, the stronger the thermal storage capacity. The fusion and freezing enthalpies of pristine PEG, MPC-Cu and PEG/MPC-Cu were studied by DSC tests, and the results are shown in Fig. 10. There were no significant peaks in the curve of MPC-Cu, meaning that MPC-Cu didn’t possess thermal storage capacity. The calculated fusion and freezing enthalpies of PEG were 183 J/g and 164.6 J/g, respectively, and those of PEG/MPC-Cu were 95.98 J/g and 87.65 J/g, respectively. The measured enthalpy of PEG/MPC-Cu was smaller than the theoretical enthalpy, which was due to the physical limitations of van der Waals forces and capillary forces between the PEG and MPC-Cu in the composite of PEG/MPC-Cu^53^. However, the physical constraints of PEG by MPC-Cu can enhance the thermal stability of the PEG/MPC-Cu composite and avoid the leakage of PEG. The pristine PEG began to melt at 59.1 °C and freeze at 37.1 °C, and in the process of melting and freezing, the peak temperatures were 62.7 °C and 34.3 °C, respectively. The composite of PEG/MPC-Cu began to melt at 54.8 °C and to freeze at 38.4 °C, and the peak temperatures in the melting and freezing process were 60.1 °C and 33.5 °C, respectively. Compared to pristine PEG, the peak temperature values of the prepared ss-PCM were lower, which could be explained by the fact that adding MPC-Cu could increase the thermal conductivity of PEG^54^.

### Thermal conductivity of PEG/MPC-Cu

**Figure 11:**
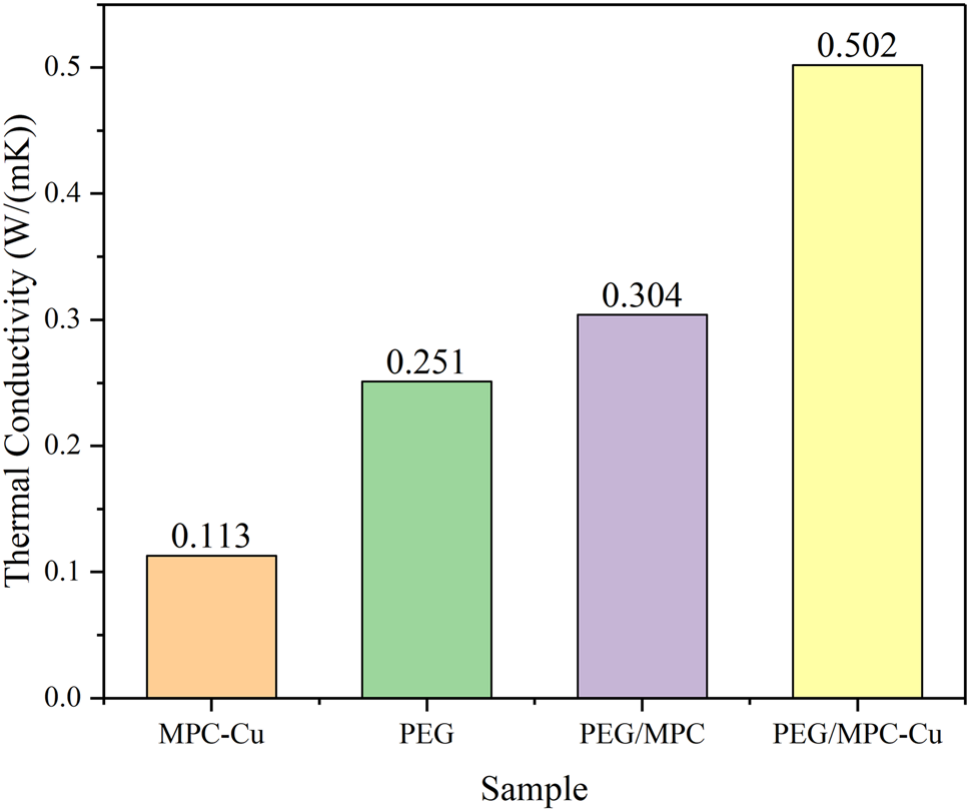
Thermal conductivities of PEG, PEG/MPC and PEG/MPC-Cu.

The thermal conductivities of PEG, PEG/MPC and PEG/MPC-Cu were tested by a TPS thermal constant analyzer, and the study results are shown in Fig. 11. Figure 11 showed that the thermal conductivity of pure PEG was 0.251 W/(m K). After PEG was embedded in MPC without copper microspheres, the thermal conductivity of PEG/MPC increased to 0.304 W/(m K)^55^, which demonstrated that immobilizing PEG into mesoporous carbon carriers was an effective measure to increase the thermal conductivity. The large surface area and the mesoporous channels of MPC-Cu benefited from the process of pyrolysis. However, as the pores of MPC-Cu were filled with air, the thermal conductivity of MPC-Cu decreased, which was only 0.113 W/(m K), and after PEG was adsorbed by MPC-Cu, PEG molecules filled the pores of MPC-Cu. The heat transfer efficiency of PEG is significantly higher than that of air; the heat transfer path occurred through the porous carbon skeletons^53^, which improved the thermal conductivity of PEG/MPC-Cu compared to PEG. Moreover, the thermal conductivity of PEG/MPC-Cu, which was 0.502 W/(m K), improved by 100% and 66.7%, respectively, compared with those of pristine PEG and PEG/MPC. Therefore, introducing copper microspheres into MPC is an effective strategy to increase the thermal conductivity of PEG/MPC-Cu.

### Comparisons between PEG/MPC-Cu and other composite ss-PCMs

As mentioned above, the fusion enthalpy and thermal conductivity are significant parameters to evaluate the practicability of ss-PCMs. The comparison results between PEG/MPC-Cu and other ss-PCMs are shown in Table 1. Although the fusion enthalpy and thermal conductivity of PEG/MPC-Cu are not as high as some of the other composite ss-PCMs, PEG/MPC-Cu is still a competitive product. Moreover, the preparation of MPC-Cu is a simple and environmentally friendly method. Therefore, the application prospect of PEG/MPC-Cu is promising.

**Table 1:** Comparisons between PEG/MPC-Cu and other composite ss-PCMs.

Sample	Fusion enthalpy (J/g)	Thermal conductivity (W/(m K))	References
PEG/MPC-Cu	95.98	0.502	This work
CPCM3	82.73	0.402	^54^
PEG/PDAM-3	133.20 ± 2.50	0.288	^37^
PEG/Dop-SF-3	73.8	Not mentioned	^56^
FS-CPCM	101.1	0.33	^57^
PEG/CMS-AC	83.2	0.62	^38^
PEG/DPMS	69.77	0.491	^58^

## Conclusion

In this study, we introduced copper microspheres into mesoporous carbon spheres by in situ reduction, and the as-prepared hybrid particles of mesoporous carbon-copper microspheres were utilized as the supporter of polyethylene glycol to synthesize a shape-stabilized phase change material. The results suggested that the adsorption and in situ reduction of metal ions used in this study effectively enhanced the thermal conductivity of ss-PCM, and the thermal conductivity of PEG/MPC-Cu was improved by 100% compared with that of pristine PEG. Moreover, the thermal storage capacity and thermal stability of PEG/MPC-Cu were favorable. Therefore, the as-prepared ss-PCM of PEG/MPC-Cu is a promising material for practical applications.
